# Psychological resilience and anxiety in response to COVID-19

**DOI:** 10.1192/j.eurpsy.2022.1232

**Published:** 2022-09-01

**Authors:** T. Ionescu, V. Boscaiu, B. Fetecau, C. Tudose

**Affiliations:** 1 University of Medicine and Pharmacy Carol Davila, Neuroscience 6, Bucharest, Romania; 2 Romanian Academy of Sciences, Institute Of Mathematical Statistics & Applied Mathematics Of Romanian Academy, Bucharest, Romania; 3 University of Medicine and Pharmacy Carol Davila, Cardiology, Bucharest, Romania

**Keywords:** Covid-19, general practitioner, pandemic, Anxiety

## Abstract

**Introduction:**

In Romania, the first case of COVID-19 was detected on 26 February 2020 and the number of cases has been rising afterward.

**Objectives:**

The goal of this study was to assess anxiety and resilience regarding the COVID-19 pandemic and to analyse possible protective measures and risk factors.

**Methods:**

This is a cross-sectional study and data were collected March and April 2021. Participants filled in the Zung Self-Rating Anxiety Scale and the Connor-Davidson Resilience Scale.

**Results:**

The sample consisted of 440 participants who presented to the general practitioner (female-65.7%) and the most representative age group was 35-64 years old; 18.4% of the participants stated that they were infected; 56.6% reported that they do not know anyone in their entourage who was infected/ has died of COVID-19 (group A), 32.9% knowing people with an infection in their close social environment (group B) and 10.5% had close people who died (group C). Almost half of the respondents (49,3%) scored above the cut-off point of the anxiety index (mild 38.6%, moderate 9.9%, severe 0.8%). As we expected, there is a strong negative correlation between anxiety levels and resilience (Pearson Correlation=-.551, p<0.01). If groups A and B had similarities regarding anxiety levels (44.97 and 44.23), those knowing someone who died of COVID-19 (group C) had a higher anxiety level (47.81%) (p<0.05).

**Conclusions:**

The recent COVID-19 pandemic has caused an understandable surge in anxiety among the general population. Low level of resilience is predictive of the phenomenon of having high anxiety in the face of the death of others.

**Disclosure:**

No significant relationships.

